# Active pulmonary targeting against tuberculosis (TB) via triple-encapsulation of Q203, bedaquiline and superparamagnetic iron oxides (SPIOs) in nanoparticle aggregates

**DOI:** 10.1080/10717544.2019.1676841

**Published:** 2019-11-06

**Authors:** Wilson Poh, Nurlilah Ab Rahman, Yan Ostrovski, Josué Sznitman, Kevin Pethe, Say Chye Joachim Loo

**Affiliations:** a School of Material Science and Engineering, Nanyang Technological University, Singapore, Singapore;; b Lee Kong Chian School of Medicine and School of Biological Sciences, Nanyang Technological University, Singapore, Singapore;; c Department of Biomedical Engineering, Technion, Israel Institute of Technology, Haifa, Israel;; d Singapore Centre for Environmental Life Sciences Engineering (SCELSE), Nanyang Technological University, Singapore, Singapore

**Keywords:** Pulmonary drug delivery, tuberculosis, superparamagnetic iron oxides, solvent emulsion, computational fluid dynamics, nanoparticle aggregates

## Abstract

Tuberculosis (TB) has gained attention over the past few decades by becoming one of the top ten leading causes of death worldwide. This infectious disease of the lungs is orally treated with a medicinal armamentarium. However, this route of administration passes through the body’s first-pass metabolism which reduces the drugs’ bioavailability and toxicates the liver and kidneys. Inhalation therapy represents an alternative to the oral route, but low deposition efficiencies of delivery devices such as nebulizers and dry powder inhalers render it challenging as a favorable therapy. It was hypothesized that by encapsulating two potent TB-agents, i.e. Q203 and bedaquiline, that inhibit the oxidative phosphorylation of the bacteria together with a magnetic targeting component, superparamagnetic iron oxides, into a poly (D, L-lactide-co-glycolide) (PDLG) carrier using a single emulsion technique, the treatment of TB can be a better therapeutic alternative. This simple fabrication method achieved a homogenous distribution of 500 nm particles with a magnetic saturation of 28 emu/g. Such particles were shown to be magnetically susceptible in an *in-vitro* assessment, viable against A549 epithelial cells, and were able to reduce two log bacteria counts of the Bacillus Calmette-Guerin (BCG) organism. Furthermore, through the use of an external magnet, our *in-silico* Computational Fluid Dynamics (CFD) simulations support the notion of yielding 100% deposition in the deep lungs. Our proposed inhalation therapy circumvents challenges related to oral and respiratory treatments and embodies a highly favorable new treatment regime.

## 1. Introduction

Tuberculosis (TB) is a contagious lung infectious disease and, despite immunization, still continues to scourge humanity. This airborne disease affects one third of the world’s population (Mehanna et al., 2014), and has climbed the ranks to distinguish itself as one of the top ten causes of mortality (W. H. Organization, 2018). TB is caused by the bacterium, Mycobacterium tuberculosis (MTB), and can manifest itself as latent or active. In the latent state, the bacteria are engulfed by the alveolar macrophages, forming a granuloma that immobilizes their activity; although bacteria proliferation is still possible (Hirota et al., 2007). The ability of an infected person to contain the disease is influenced by his/her health, i.e. age, infection with Human Immunodeficiency Virus (HIV), smoking, etc., which can compromise the structural integrity of the granuloma and liberate the sojourned bacteria. Patients diagnosed with active TB, on the other hand, will experience the typical symptoms of cough, fever, blood-tinged sputum, and weight loss.

Typically, TB is treated orally with a cocktail of four first-line drugs, namely Rifampicin, Isoniazid, Pyrazinamide, and Ethambutol. Treatment usually lasts for a period of 6–9 months, followed by a post treatment of 1–2 years (Pham et al., 2015). However, because of prolonged drug usage, liver and kidney damage may be sustained. To address this, other treatment strategies have been proposed, including targeting drugs directly to the infectious site; for instance, through the pulmonary route (Gandhimathi et al., 2015). The respiratory tract has an enormous surface area for air-exchange, which can be exploited for drug targeting. In addition, unlike oral therapy, targeting drugs to the lungs avoids the first-pass metabolism, which will likely increase drug bioavailability, and may potentially reduce the cost and duration of treatment. Currently, there exists a plethora of pulmonary drug delivery systems composed of biocompatible polymers, such as chitosan (Grenha et al., 2005), PLGA (Makino et al., 2004; Ohashi et al., 2009; Hirota et al., 2010), or liposomes (Chimote & Banerjee, 2010) that can be used for this purpose. However, many of these still largely remain in the preclinical phases of development, with only a few currently available on the market.

Delivering drugs through the pulmonary route, however, has its own set of challenges (d'Angelo et al., 2015). For one, commercially-available aerosolizing platforms, i.e. nebulizers, pressurized Metered Dose Inhalers (pDMIs), and Dry Powdered Inhalers (DPIs), have astonishingly low deposition efficiencies (O'callaghan & Barry, 1997; Labiris & Dolovich, 2003; Pitance et al., 2010). It is known that the aerodynamic size of the aerosolized particle, and other geometrically-related factors, do affect deposition mechanics. Particles with an aerodynamic size greater than 5 µm are usually impacted in the oropharyngeal region, while particles in the size range of 1–5 µm yield deep lung delivery via sedimentation (Gandhimathi et al., 2015; Rashid et al., 2015). Besides geometrical factors, extrinsic influences, for instance, through a magnetic field can also aid in increasing deep lung delivery. In a recent study (Ostrovski et al., 2016), it was shown that water droplets, loaded with superparamagnetic iron oxides (SPIOs), in the size range of 0.5–3.0 µm can achieve deposition of up to 100% in the acinar region, under an applied magnetic field. SPIOs can therefore be an attractive proponent to develop magnetically-driven drug carriers because they are biocompatible and respond instantaneously in the presence of a magnetic field, to within a distance of 10–15 cm (Arruebo et al., 2007).

Here, we hypothesized that co-encapsulating SPIOs along with the novel anti-TB agents (i.e. Q203 and bedaquiline (BDQ)) in combination into 500 nm–sized drug carriers, allows for improved pulmonary delivery and more effective killing of TB bacteria than free drugs. BDQ is a diarylquinoline bactericidal antibiotic approved for the treatment of TB, and is specifically used for Multidrug-Resistance Tuberculosis (MDR-TB). Q203 is an imidazopyridine amide (IPA) compound that inhibits bacteria growth by impeding the oxidative phosphorylation at the respiratory cytochrome bc_1_ complex (Pethe et al., 2013). In this paper, we report on the fabrication of 500 nm spherical poly (D, L-lactide-co-glycolide, 50:50) (PDLG) drug carriers loaded with SPIOs, Q203 or BDQ (or in combination), through a single emulsion technique. This technique is a one-pot synthesis process that produces particles with a narrow size distribution. At this size range, particles can traverse through the refuse of bifurcating airways of the lungs (Heyder, 2004), and when combined with a magnetic component allows for deposition in the acinus (Ostrovski et al., 2019). PDLG was selected because it is biodegradable, biocompatible, and possesses tunable drug release profiles. We also present its drug release kinetics, toxicity to A549 lung epithelial cells, the killing efficacy on Bacillus Calmette-Guerin (BCG), and its deposition potential in lungs using numerical simulations.

## 2. Methods and materials

### 2.1. Materials

Ammonium hydroxide solution (28.0–30.0% NH_3_ basis), dimethyl sulfoxide (DMSO) (99.9%), dichloromethane (DCM), poly (vinyl alcohol) (PVA) (87–90% hydrolyzed, average mol. wt. 30,000–70,000), oleic acid (OA), iron (II) chloride tetrahydrate (≥99.0%), and iron (III) chloride hexahydrate crystallized (98.0–102%) was obtained from Sigma Aldrich (Singapore). The PDLG 5010 polymer was purchased from Corbion Purac (Singapore). Q203 and BDQ were a kind gift from Dr. Kevin Pethe, Lee Kong Chian (LKC) School of Medicine, Nanyang Technological University.

### 2.2. Fabrication of the SPIOs

The SPIOs were produced via a co-precipitation of iron (II) and iron (III) salts in an alkaline environment. The fabrication protocol follows that of Xie et al. (Xie et al., 2010). Briefly, 0.30 g of FeCl_2_ and 0.82 g of FeCl_3_ were dissolved in 20 mL of DI-water that has been deoxygenated, and were heated to 80˚C under nitrogen purging. The solution was heated for 30 min before 1 mL of ammonium hydroxide was added, drop-wise, and was heated under the same temperature for another 30 min. 1 mL of oleic acid (OA) was then added to the black precipitate and the temperature was raised to 95˚C to be heated for 90 min. The resulting mixture was cooled, centrifuged, and washed thrice before storing in DCM.

### 2.3. Fabrication of the pulmonary drug carrier

The pulmonary drug carrier was fabricated using a single emulsion (O/W) technique. The oil phase (O) consisting of 200 mg of PDLG 5010, 1.0 mg of Q203, 5.1 mg of BDQ, 100 mg of SPIOs, and 10 mL of DCM were homogenized at 19,000 rpm with 20 mL of 6% PVA solution (W) for 10 min. The resulting emulsion was poured into a beaker containing 250 mL 0.6% PVA solution and then overhead stirred for three hrs at 400 rpm to evaporate off the DCM. Once the DCM has evaporated, the resulting particles were then centrifuged, decanted, and washed thrice with DI-water before they were freeze-dried.

### 2.4. Characterization of the drug carrier

#### 2.4.1. Size and morphology of the SPIOs and drug carrier

The surface morphology and the size of the drug carrier were analyzed using a Field Emission Scanning Electron Microscope (Fe-SEM) (JEOL JSM-6340F SEM) at an operating voltage of 5 kV and a probe current of 12 µA. The sample for analysis was prepared by spreading the freeze-dried drug carriers onto a carbon tape. The sample was then coated with platinum at 20 mA for 60 secs. The size of the SPIOs and the dispersion of the SPIOs within the drug carrier were observed using a Transmission Electron Microscope (TEM) (Carl Zeiss Libra 120 Plus) at an operating voltage of 120 kV. The samples to be analyzed were dispersed in DI-water, sonicated for 10 min, and then pipetted out onto a copper grid and left to dry for 24 hrs.

#### 2.4.2. Dynamic light scattering (DLS)

The average particle size and polydispersity was measured using a Malvern Nanosizer. The drug carrier was suspended in DI-water, sonicated for 10 min, and then immediately pipetted out into a cuvette for analysis. The measurements were done in triplicates.

#### 2.4.3. Magnetic saturation of the SPIOs and the drug carrier

The magnetic saturation for both the SPIOs and the drug carrier was determined using a Vibrating Sample Magnetometer (VSM) (VSM Lakeshore 7400) at a maximum magnetic field of 10,000 Gauss (G). The samples were prepared by placing five mg of the dried SPIOs and drug carrier, separately, into a polytetrafluoroethylene (PTFE) tape. The samples were then place in the sample holder for analysis.

#### 2.4.4. Numerical simulations

Computational Fluid Dynamics (CFD) simulations were performed using a commercial finite volume method (FVM) solver (ANSYS Fluent) to simulate magnetic particle inhalation into an acinar geometry. The geometry mimics the alveolar environment and consists of 372 individual alveoli that densely populate the space surrounding a branching airway tree, starting with one inlet and bifurcating a mean of 4.38 generations, with up to 8 generations (see Hofemeier et al. for further details (Hofemeier et al., 2018)). Breathing was modeled through a self-similar motion of the walls of the acinar domain, following a sinusoidal volumetric inflation function with an amplitude of 16.8% off the resting volume (corresponding to functional residual capacity) and a breathing cycle of three secs.

Physics of fluid (i.e. air) motion are described by the transient Navier-Stokes’ equations along with the continuity equation, where the inhaled air is assumed to be Newtonian, incompressible and isothermal, with density ρ = 1.22 kg/m^3^ and viscosity μ = 1.79·10^−5 ^ Pa·s. A no-slip boundary condition is implemented on the moving geometry walls, whereas the domain inlet (i.e. outlet during deflation) is defined as a pressure-inlet, which results in air being drawn into the geometry as it inflates, and pushed out as it deflates.

An in-house discrete element method (DEM) solver was used to simulate the motion of the particles (Ostrovski et al., 2016, 2019). Particles were one-way coupled to the flow and their motion was solved according to Newton’s force balance in a Lagrangian framework, where gravitational, drag and Brownian forces were considered. The direction of the gravity was previously shown to have little effect on the particle deposition results (Hofemeier et al., 2018), and was arbitrarily directed here in the –y direction. Particles with density of ρ_p_ = 1490 kg/m^3^ and diameter of 0.5 mm are injected into the domain via the inlet after 0.6 secs to account for the lung’s dead space (i.e. crossing through the conducting airways) until 1.5 secs (i.e. peak inhalation). Upon contacting a wall, particles are assumed to have deposited. Briefly, following rigorous mesh convergence tests, a total of 4.25 × 10^6^ tetrahedral cells were used to mesh the geometry, whereas a time step of 10^−3^ secs was used for the flow and 2·10^−7^ secs for the particles, and 10,000 particles were injected to assure accurate deposition statistics.

#### 2.4.5. *In-vitro* magnetic targeting

**Figure 1.**
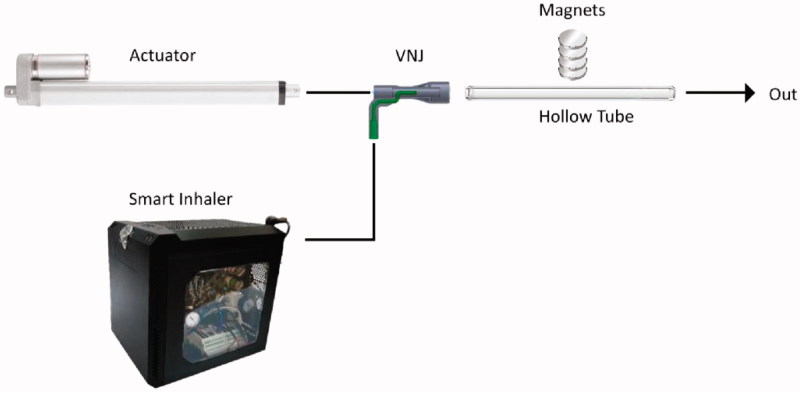
Schematic of the *in-vitro* magnetic targeting setup.

A set-up to assess the magnetic targeting of the drug carriers was erected (Figure 1). The components of this setup consist of a 16.8 mm hollow tube mimicking an adult trachea, a Ventilator-Nebulizer Junction (VNJ), a smart inhaler system (custom-made nebulizer coupled with an electronic control that releases short pulse of aerosol bolus), an actuator (LEY25B-300-S16P1, SMC), and a stack of permanent magnets. The smart inhaler and the actuator were connected to the inlet of the hollow tube by means of a VNJ and the outlet of the tube was connected to a chemical hood. A stack of four permanent magnets were placed outside of the tube approximately at the center (lengthwise) of the tube. The magnetic targeting capability of the drug carrier was assessed by passing an aerosolized brine solution (DI water:NaCl (10:1)) of the drug carriers through the hollow tube and having the magnets along the tube to arrest the particles. A breath-hold (BH) of five secs was calibrated to initiate after the aerosol bolus was directly beneath the magnet. The suspension was aerosolized by a smart inhaler which releases short and controlled pulse of the aerosol. The flow of 1.145 L/min was generated by a linear actuator. The magnets are four disc-shaped (30 mm in diameter, 15 mm in height) rare-earth permanent magnet separated by 5 mm thick plastic plates. The response of the aerosolized suspension in relation to the magnet was also assessed. For this assessment, the magnet’s distance in relation to the tube was varied from 0 to 10 mm and finally to 20 mm.

#### 2.4.6. Drug encapsulation efficiency (EE)

The drug encapsulation efficiency was determined using Liquid Chromatography Mass Spectroscopy (LC-MS) (Agilent LC 1260 MS 6120). The mobile phase consists of acetonitrile and DI-water (50:50) with 0.1% v/v of formic acid, using Zorbax RX-C8 (150 mm x 4.6 mm) as the stationary phase. The samples were maintained at 25˚C and injected into the column with a flow rate of 1 mL/min and analyzed at an m/z of 279 for Q203 and m/z of 555 for BDQ. 20 mg of the drug carrier was dissolved in DMSO and centrifuged at 13,000 rpm for five min. The supernatant was then collected for analysis. The drug encapsulation efficiency was carried out in triplicates and is expressed as the percentage of actual drug loaded over the initial amount of drug (mg).

$$\text{Encapsulation efficiency }\left(\%\right)=\;\frac{\text{Actual drug loaded }\left(\mathrm{mg}\right)}{\text{Initial amound of drug }\left(\mathrm{mg}\right)}\times \;100\%$$

#### 2.4.7. Drug release studies

10 mg of the drug carrier was placed inside a 2 mL microtube containing 1 mL of Simulated Lung Fluid (SLF) and shaken at 100 rpm at 37˚C. The drug release studies were conducted for 24 hrs in triplicates. At pre-determined time intervals, the release media was pipetted out, transferred to a new microtube, and then frozen in the −20˚C refrigerator. The microtube was then freeze-dried before 1 mL of DMSO was added. The samples were analyzed using LC-MS with the same settings used for the encapsulation efficiency studies.

#### 2.4.8. Cell viability

A549 cells (10,000/100 μL) were seeded onto 96-well plates and incubated overnight at 37˚C under 5% CO_2_ atmosphere. The cells were maintained in high-glucose Dulbecco’s Modified Eagle Medium (DMEM) containing 10% fetal bovine serum (FBS, v/v), and 1% penicillin/streptomycin (P/S, v/v). After incubation, the cells were replenished with 100 μL of fresh media containing 100–500 μg/mL of the drug carrier with and without SPIOs (both carrier types without the inclusion of drugs), and incubated for 24, 48 and 72 hrs. And at each time point, the media was removed, and 100 µL of PrestoBlue assay that has been diluted down ten-folds was added. The cells were then incubated for another hour. After that, 50 µL of the media was pipetted out onto new 96-well plates and analyzed while the remaining media was discarded and replenish with fresh media. The control used for this analysis was A549 cells without drug carrier. The fluorescence readings were done using a plate reader (Tecan Infinite M200) at 570 nm and 600 nm.

#### 2.4.9. Assessment of the number of viable bacteria

BCG bacteria at OD 0.005 in the mid-log phase were grown in 7H9 broth with glycerol to a 10^6^ CFU count. MIC_99_ of the single encapsulated Q203 and BDQ, and the combination encapsulated Q203 and BDQ were introduced the BCG bacteria once at the beginning of the experiment. The untreated BCG and the drug carrier without drugs were used as controls. At day 0, 4, 10, and 15, 50 μL aliquots of the culture was dropped onto agar plates and allowed to incubate at 37˚C with 5% CO until bacteria colonies were formed. The MBC was determined by multiplying the visible colonies by the dilution factor.

## 3. Results

### 3.1. Fabrication of SPIOs and drug carrier

**Figure 2.**
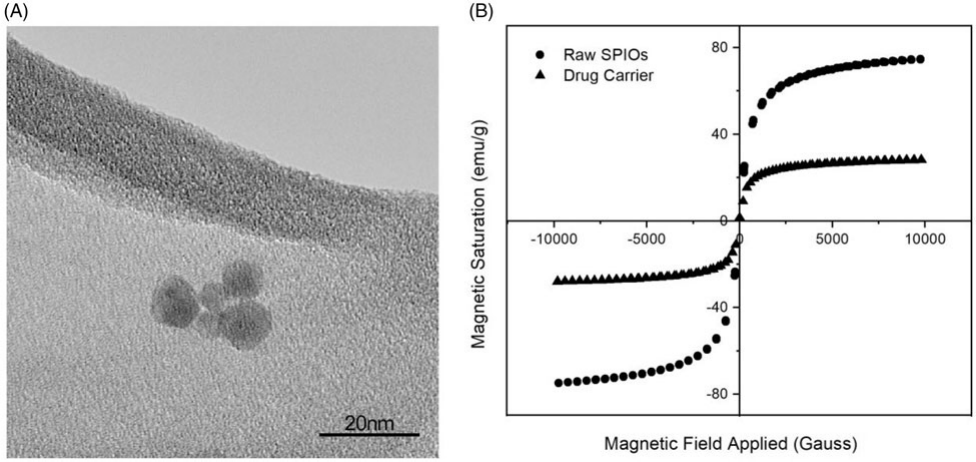
Characterization of SPIOs. (A) TEM image of the SPIOs. (B) Hysteresis curve of the raw SPIOs and the drug carrier using VSM.

The drop-wise addition of ammonium hydroxide into iron (II) and iron (III) salts solution yielded spherical SPIOs with a narrow size distribution of 9 ± 3.92 nm as shown in Figure 2(A). These SPIOs possess superparamagnetic properties with no remanence and coercivity, upon the removal of the magnetic field. The SPIOs possess a high magnetic saturation of 74.8 emu/g, and when encapsulated into PDLG sub-micron particles (the drug carrier), a drop in the overall magnetic saturation to 28.0 emu/g was observed (Figure 2(B)), due to the shielding effect of the polymer. This magnetic saturation value is subsequently applied in the CFD simulations to validate the deposition profile of these drug delivery particles in the acinus.

**Figure 3.**
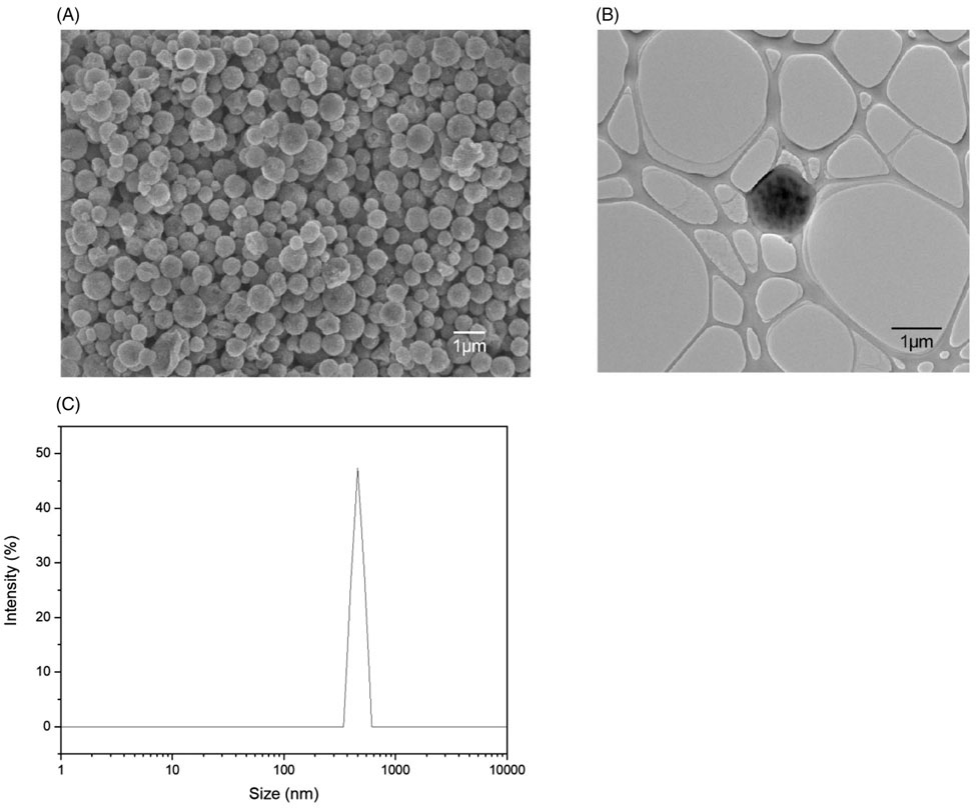
Characterization of the drug carrier using different characterization techniques. (A) FE-SEM image at 8000x magnification. (B) TEM image at 4000x magnification. (C) DLS spectrum of the drug carrier having a mean hydrodynamic diameter of 476.9 nm ± 28.7 and a PDI of 0.51.

Next, PDLG drug carriers, encapsulating SPIOs, were synthesized through a single emulsion technique that yielded uniformly-sized, spherical particles with a smooth morphology of around 500 nm, as shown in Figure 3(A). From the TEM image in Figure 3(B), the contrast of the PDLG carrier and the SPIOs can be seen with the bulk of the SPIOs located at the core of the carrier. DLS (Figure 3(C)) measurements confirmed a mean hydrodynamic diameter of 476.9 ± 28.7 nm with a Polydispersity Index (PDI) of 0.51.

### 3.2. *In-silico* deposition efficiency

**Figure 4.**
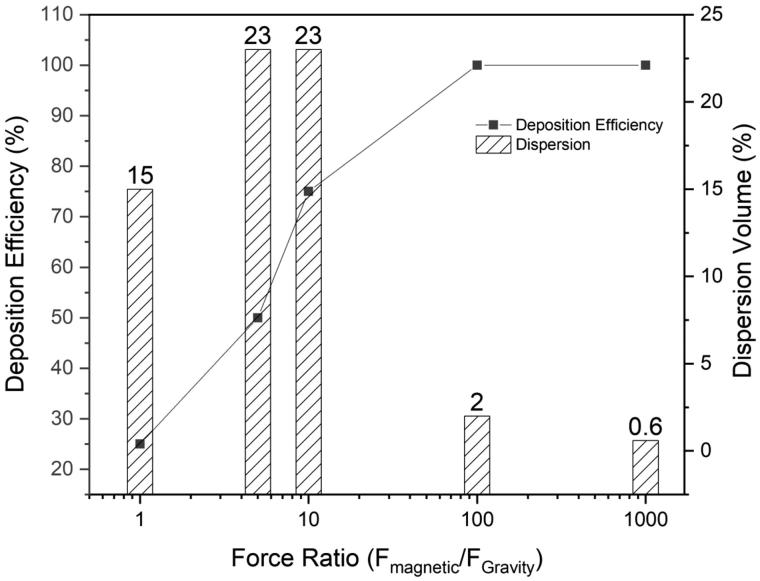
Deposition efficiency of the SPIOs-encapsulated carriers as a function of the force ratio.

To validate the deposition efficiency of the pulmonary delivery system, CFD simulations were performed with 10,000 carrier particles (500 nm in diameter, 28 emu/g magnetic saturation) in the acinus to analyze the deposition efficiency of these particles with respect to the ratio of the magnetic field applied (F_magnetic_) and gravitational pull (F_Gravity_). Figure 4 shows that as the force ratio (F_magnetic_/F_Gravity_) increases, the deposition efficiency of the particles into the acinus increases. When the magnetic field yields a force ratio ≥ 10, the particles can be deposited in the acinus with little or no loss yielding high deposition efficiency. However, as the magnetic field increases, the dispersion of the aerosol carriers throughout the acinar region suffers (see Supplementary Figure 1 in which the simulations explore the influence of different force ratios). When the force ratios were at 5 and 10, dispersion of the particles spans across 23% of the acinar volume. However, as the force ratio increases from 10 to 1000, the dispersion volume (i.e. the volume covered by the particles in relation to the entire acinar volume) decreases from 23% to 2% and then eventually to 0.6%, i.e. one alveolus. Higher force ratios, i.e. 100 and 1000, are unsuitable as a wider particle-drug distribution within the lungs is always preferred, especially for TB treatment.

### 3.3. *In-vitro* magnetic targeting

**Figure 5.**
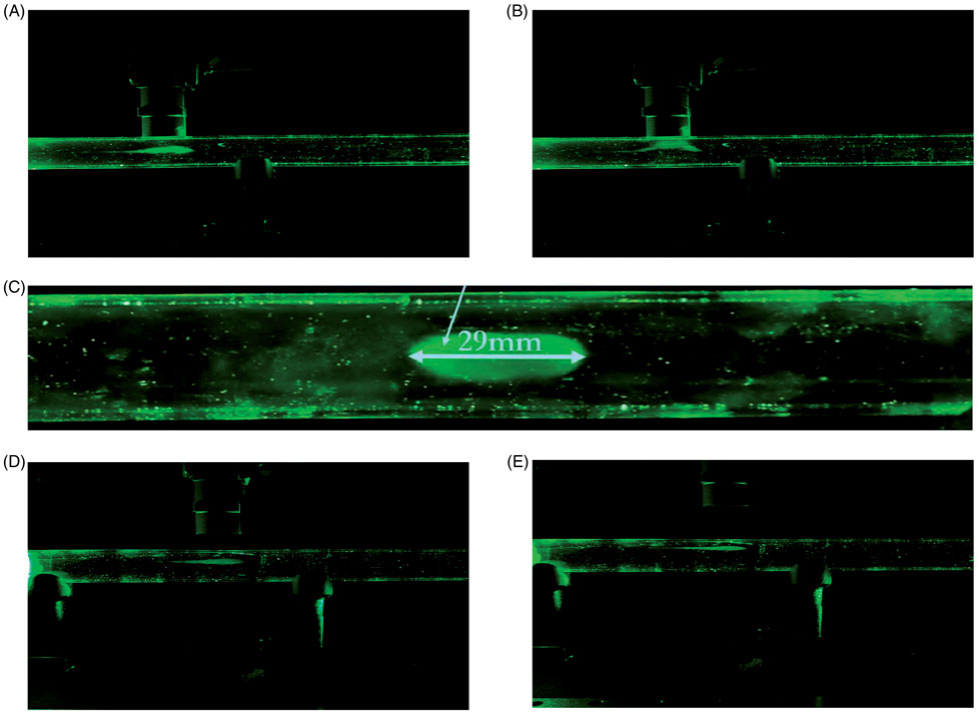
*In-vitro* magnetic targeting. (A) Drug carriers aerosolized with a brine solution of 10:1 DI water:NaCl into a 16.8 mm inner diameter hollow tube. The airflow was set at 1.145 L/min. The magnet was placed 0 mm from the hollow tube. The aerosol bolus was calibrated to stop directly below the magnet. (B) The aerosol bolus was attracted towards the magnet. (C) The deposited aerosol bolus onto the inner wall of the hollow tube. The elliptical width was measured at 29 mm. (D) Experiment with magnet placed 10 mm from the hollow tube. (E) Experiment with magnet placed 20 mm from the hollow tube.

Aerosolization of the drug carriers suspended in the brine solution was generated via a modified nebulization setup and measured at a Mass Median Aerodynamic Diameter (MMAD) of 0.4506 ± 0.0302 µm. After aerosolization, the aerosol bolus produced follows the airflow in and travels along the hollow tube until it reaches beneath the magnet, during which a BH of five secs was activated (Figure 5(A)). The BH was simulated through the cessation of the airflow into the hollow-tube. It can be seen in Figure 5(B) that shortly after the breath-hold, the particles were drawn up towards the magnet. Only a small remainder of the nebulized particles was ‘exhaled’. A clear elliptical stain was visible when the magnet was removed from its position (Figure 5(C)). Since not all the deposited particles were localized in one area, the deposition ratio (i.e. the ratio of the particles deposited near the magnet to the particles deposited elsewhere is infinity) cannot be precisely quantified. The experiment was repeated again, varying the magnet’s distance from the tube to 10 mm and then 20 mm. Similarly, for both these cases, the aerosol bolus was again seen to be arrested by the magnet (Figure 5(D,E)).

### 3.4. Drug encapsulation efficiency and release profiles

The encapsulation efficiencies of Q203 and BDQ were 55% and 57%, respectively, as shown in Table 1. Both Q203 and BDQ have high log P values which are compatible with the hydrophobic nature of the PDLG polymer, and enable them to dissolve in DCM to form the oil phase (with SPIOs and polymer). A slight reduction in the encapsulation efficiencies for both Q203 and BDQ are observed when both drugs were loaded in combination, likely due to competitive loading of the two drugs within a single carrier.

**Table 1.** Encapsulation efficiency of the Q203 and bedaquiline drug in PDLG 5010.

	Log P	Encapsulation efficiency (%)
Q203 only	7.62*	54.5 ± 0.7
BDQ only	7.52	56.8 ± 2.9
Q203 + BDQ (combination)		40.6 ± 1.6 (Q203)
		49.9 ± 0.8 (BDQ)

* c log P.

**Figure 6.**
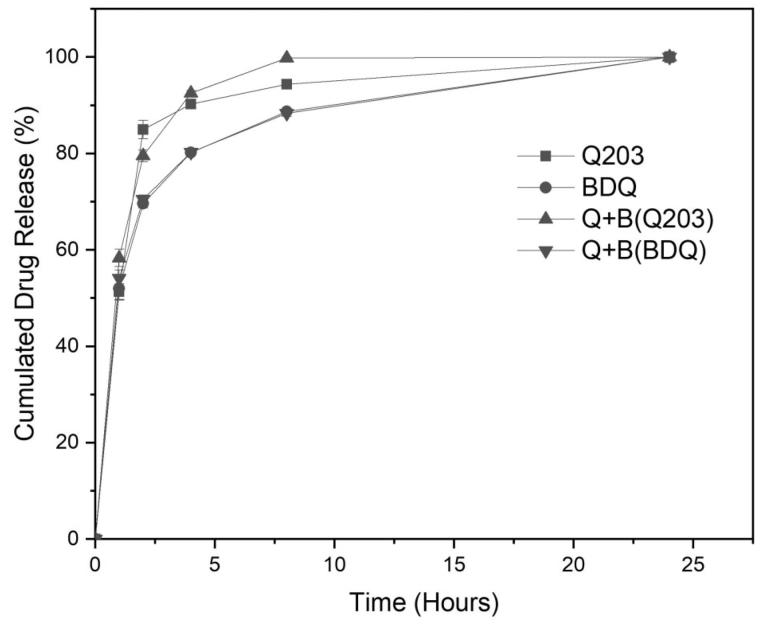
Release profiles of encapsulated Q203 only, BDQ only, and Q203 and BDQ (Q + B) in 1 mL of SLF.

Figure 6 shows the release profiles of encapsulated Q203 only, BDQ only, and the combination of Q203 and BDQ in SLF. For all three delivery systems, the release profiles exhibit similar characteristics. In the first phase (0–8 h), the drug(s) was (were) released precipitously, with a slower release in the second phase. The percentage cumulative drug remaining for all three delivery systems when plotted against time corresponds to a first-order release kinetics. This indicates that the release is concentration dependent and diffusion controlled (R^2^ ≥0.9443). Release of either drug is also independent of the other as the release kinetics are similar whether encapsulated singly or in combination. After 24 hrs, all drugs were completely released.

### 3.5. Cell viability

Cell viability was evaluated using a Prestoblue assay to determine the toxicity of these carriers on the lung epithelium cells (i.e. A549). These are type II pneumocytes that are responsible for secreting lung surfactants in the alveoli, and were used because they are one of the first cells to be exposed to the delivery system. Also, A549 is one of the *in-vitro* cell lines accepted for cytotoxicity assays (Ungaro et al., 2012; Chuan et al., 2013; Janer et al., 2014) in the lungs. Cell viability was investigated across different concentrations up to 500 μg/mL (Supplementary Figure 2). Although it is unlikely that the cells would be exposed to the particles at such high concentrations, we sought to challenge the cells to mimic a situation where the particles would accumulate in the lungs. The results demonstrate that there is no significant difference in cell viability for the advocated drug carrier with and without SPIOs. This indicates that the carrier is non-cytotoxic and may be suitable as a pulmonary delivery system, which is in agreement with other cytotoxicity studies of PLDG (Tripathi et al., 2010; Mehanna et al., 2014; Ali et al., 2015) and SPIOs (Ling et al., 2012; Schleich et al., 2013).

### 3.6. Bactericidal effect of free drugs and drug delivery system on BCG

**Figure 7.**
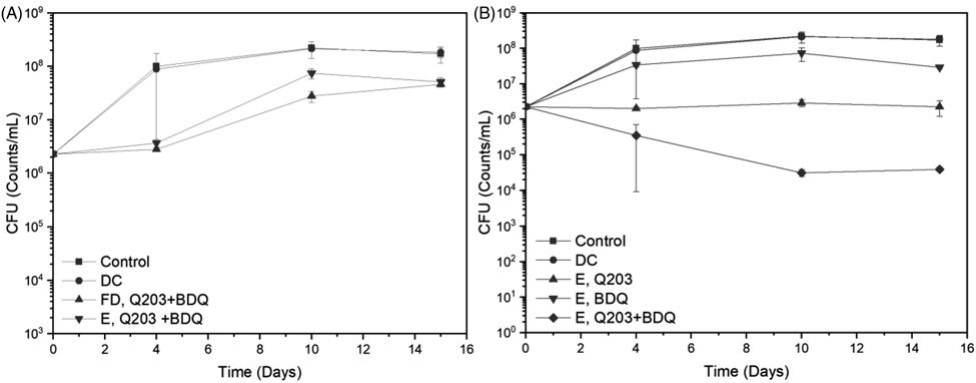
Minimum Bacterial Concentration (MBC) studies of Wild-Type (WT)-BCG against Q203 and/or bedaquiline (BDQ). Time points were set at day 0, 4, 10, and 15. (A) Comparison of the combination free drugs and encapsulated drugs at MIC_50_. (B) Comparison between encapsulated Q203 only, BDQ only, and a combination Q203 and BDQ at MIC_99_.

Here the efficacy of Q203 only, BDQ only, and the combination of both of them were individually evaluated against wild-type (WT) BCG. To ascertain the activity of the free drug combination and the encapsulated drugs, a bactericidal assessment with these two components at MIC_50_ (3 nM for Q203, 120 nM for BDQ) were evaluated. From Figure 7(A), it can be seen that there is insignificant difference in the efficacy between free drugs and the encapsulated ones, indicating that the delivery system does not interfere with the antimicrobial properties of the drugs. With this, the comparison between single and dual encapsulated drug formulation at MIC_99_ (100 nM for Q203, 500 nM for BDQ) were evaluated. For this assessment, the dosage of the drugs was increased to observe the bactericidal activity below the inoculum value of the combination. The controls used for this study are the untreated WT-BCG and the drug carrier without drugs (DC). In Figure 7(B), the single encapsulated Q203 is able to deter the growth of BCG consistently throughout the study. However, for the single encapsulated BDQ, the BCG is able to proliferate for up to 10 days (although to a lesser extent than that of the controls) before decreasing slightly at day 15. Individual encapsulated drugs, i.e. Q203 or BDQ, were unable to lower the CFU count. However, when both drugs were combined, a two-fold decrease in the bacteria colony can be observed. For the first 10 days, there was a steady decline in the bacteria colonies. After which from day 10 to 15, the killing plateaus.

## 4. Discussion

The one-pot synthesis of the drug carriers using a single emulsion technique produced mono-dispersed particles of 500 nm. The SPIOs endowed the drug carriers with magnetic properties, although a decrease in the magnetic saturation of these carriers was observed when compared to naked SPIOs. The combination of the SPIOs and the PDLG carrier was assessed for its toxicity and from the Prestoblue assay, the drug carrier was found to possess low toxicity to lung epithelial cells as shown from our *in-vitro* data (Supplementary Figure 2).

The rationale for using 500 nm particles as the size range for this pulmonary delivery was based on the deposition statistics generated by the International Commission on Radiological Protection (ICRP) (Supplementary Figure 3). This statistical model was designed according to the semi-empirical equations derived from the deposition data in humans using mono-dispersed particles (Sznitman, 2013). In this model, particles in the size range of 500 nm were shown to have the lowest total deposition in the lungs, attributed to the unresponsive nature to Brownian diffusion or gravity. Such particles introduced into the respiratory tract follow the inhalation airflow in and out of the lungs without being deposited. However, to achieve acinar delivery and avoid unintentional targeting to other organs along the respiratory tract, these carriers were incorporated with magnetically responsive SPIOs, and a large volume of deep lung deposition may thus be observed (Figure 4). Ostrovski et al. (2016) had previously demonstrated *in-silico* that when combined with a magnetic component and an appropriate breath-hold maneuver, the 500 nm water droplets can be leveraged for a targeted delivery to achieve 100% deposition in the acinus, which is similarly observed for our 500 nm PDLG carriers encapsulated with SPIOs. As seen in Figure 4, when the external field strength increases, the percentage of the deposited particles also increases. At a force ratio (F_magnetic_/F_Gravity_) more than 10, the particles were able to reach 100% deposition, but at the expense of volume dispersion (Supplementary Figure 1). An optimal balance between these two components, i.e. deposition and dispersion was found when the force ratio is at 10. At this ratio, 75% of the particles that were inhaled were deposited while achieving a dispersion volume of 23% throughout the acinus. Although, a 100% dispersion is always desired, 23% of dispersity still covers a substantial area in which the bacteria can occupy. This force ratio was therefore favorable for a large deposition, while mitigating drug wastage with a good dispersity.

To validate the above numerical analysis results, these 500 nm drug carriers were next evaluated in an *in-vitro* magnetic targeting set up. The carriers were suspended in a brine solution and nebulized into an aerosol mist before injecting into a hollow tube. The aerosol bolus that entered the tube was set at a breathable range of MMAD 0.4506 ± 0.0302 µm and was observed to be ‘captured’ beneath the external magnetic source. Within two secs of BH, the majority of the aerosol bolus was pulled towards the magnet, while the remainder followed the airflow out of the tube. The BH simulation was necessary to achieve targeting because it allows time for the particles to get pulled towards the magnet. In an experiment with no BH activated, no deposition of the aerosol bolus onto the walls of the tube was observed. To further validate the effectiveness of this magnetic-targeting ability of the drug carriers, the distances between the magnet and the tube were increased to a distance 10 mm and 20 mm. In both scenarios, the carriers continued to exhibit similar flow and ‘capture’ characteristics of the earlier experiment, confirming the magnetization capability of these active-targeting, sub-micron (500 nm) drug carriers.

Conventionally, TB is treated with Rifampicin, Isoniazid, Pyrazinamide, and Ethambutol. A combination of drugs is used so as to mitigate the evolution of MDR strains through monotherapy. On this basis, any treatment of TB should also be aligned towards multidrug therapy. In order to pair Q203 with another anti-TB agent, the accompanying drug should present no cross resistance with Q203 and also targets the ATP synthase complex in the bacteria oxidative phosphorylation, for synergy. BDQ is therefore one such candidate. From the drug release studies in Figure 6, it was observed that both drugs were released at the same rate (0.07 hr^−1^) from the drug carrier. This would allow for a fixed ratio of the two drugs to be administered. The mycobactericidal activity of the combination of Q203 and BDQ on WT-BCG is advocatory of their ability to eradicate the TB bacteria upon deposition. Individually, the drugs were unable to reduce bacteria colonies. This is because while Q203 was able arrest the growth of replicating bacteria by inhibiting the cytochrome-bc_1_:aa_3_ pathway in the third complex of the Electron Transport Chain (ETC), the non-replicating bacteria were able to sustain their viability due to an alternate branch (ctyochrome-bd) in the same complex that compensate the flow of electrons (Kalia et al., 2017). BDQ, which depletes the ATP production in the bacteria, was only able to partially kill off the replicating and non-replicating BCG. It is only when both drugs were combined that the bacteria log count reduced by two folds. Q203 and BDQ inhibitory capabilities to disrupt the flow of electrons in the ETC are crucial factors to the effectiveness of this delivery system.

A comparison was made with other promising pulmonary platforms to evaluate the efficacy of this delivery system. It is important to note that unless the conditions in which the experiments evaluated were similar, the comparison cannot be done impartially. A study done by Keiji Hirota et al. (Hirota et al., 2010) to examine the bactericidal effects on Rifampicin-loaded PLGA microspheres (RPF-PLGA MS) on BCG-infected alveolar macrophages (NR8383). In that study, the administration of RPF-PLGA MS on the BCG-infected macrophages had a greater mycobactericidal effect than un-encapsulated ones and was able to reduce one and a half log CFU counts of the BCG (initial count of 10^5^) with an incubation for four days; and an approximately two log reduction with an incubation of seven days. The mycobactericidal effect was more pronounced in encapsulated Rifampicin because after being engulfed into the macrophages, the drug accumulated within them through the sustained released of microspheres resulted in a larger amount of Rifampicin than un-encapsulated ones. Another study conducted by Sandra Suarez et al. (Suarez et al., 2001) explored the effects of PLGA microspheres containing Rifampicin (R-PLGA MS) on MTB. In this *in-vitro* study, the growth of 50% of the MTB was inhibited when 2 µg/mL of the R-PLGA MS was introduced while a 100% was observed when higher concentrations (20 and 100 µg/mL) were used. In both studies, although their delivery systems were capable of bactericidal activities, there was no evidence to support the capability of a high deposition or targeting into the lungs. The results obtained from our newly developed pulmonary delivery system highlight both the bactericidal synergism between Q203 and BDQ and the communicative ability to achieve high deposition amounts into the lungs, as shown from our *in-vitro* assays and CFD simulations. The combination of these elements together with the low cytotoxicity of these drug carriers substantiates our claims that this delivery system could be a potential delivery system for pulmonary delivery for the treatment of TB.

## 5. Conclusion

The encapsulation of Q203, BDQ and SPIOs into an inhalable PDLG carrier is a promising strategy against active TB. The carrier has demonstrated its ability to be targeted into lungs with high deposition volumes through *in-silico* computations and an *in-vitro* magnetic targeting assessment, have a safe interaction with A549 epithelial cells, and possesses a potent killing efficacy on BCG. This pulmonary delivery system has proven to be comparable in terms of the mycobacterial activity with other existing pulmonary systems, and further addresses the issue of the low deposition efficiencies from aerosolizing platforms which other systems lack. In addition, the method of fabrication is straightforward, using a single emulsion technique to incorporate all the components into the drug carrier.

## Supplementary Material

Supplemental Material
