# Supplementary material for: Active pulmonary targeting against tuberculosis (TB) via triple-encapsulation of Q203, bedaquiline and superparamagnetic iron oxides (SPIOs) in nanoparticle aggregates
Source: Drug Deliv. 2019 Nov 6;26(1):1039–48. doi: 10.1080/10717544.2019.1676841 (PMC6844420; doi:10.1080/10717544.2019.1676841)
Supplement: Supplemental Material [file IDRD_A_1676841_SM3487.docx]

**Supplementary Figures**


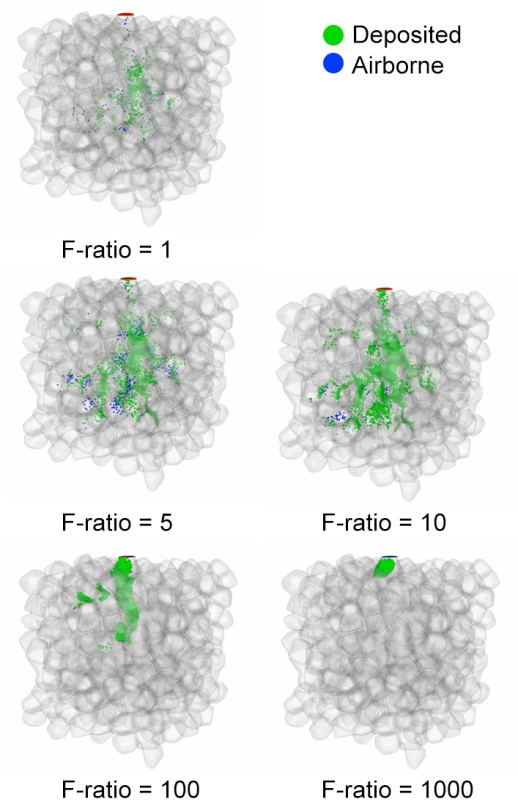


Suppl. Fig. 1 Simulation of 372 alveoli in the acinus. 10,000 particles of size 500 nm with a magnetic saturation of 28 emu/g were introduced into the acinar model with a breathing cycle and subjected to different force ratios (magnetic force against gravitation force) of 1, 5, 10, 100, and 1000. As the magnetic component in the force ratio increase, the deposition amount also increases but reduces the dispersion.

**
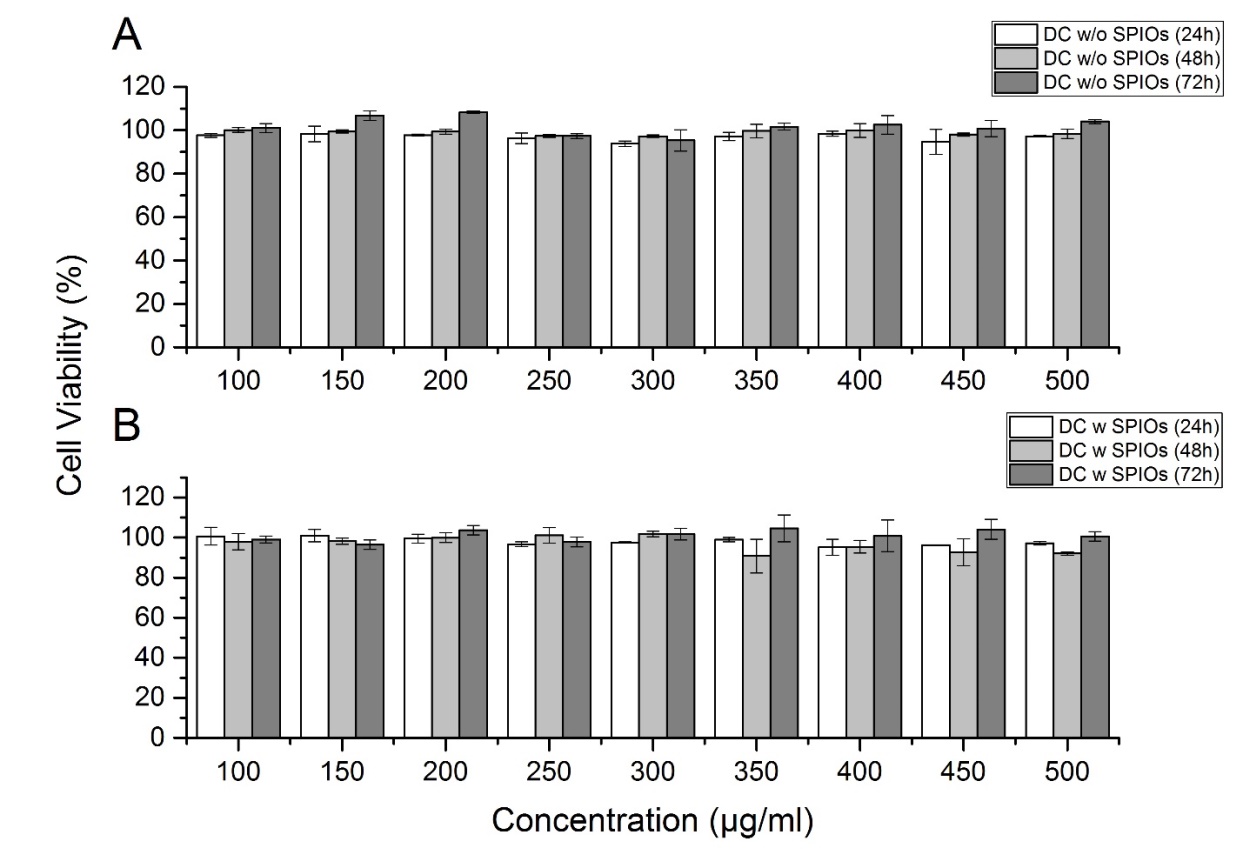
**

Suppl. Fig. 2 A549 cell viability measured by Prestoblue assay against (A) drug carrier (DC) without SPIOs at 24, 48, and 72h, (B) drug carrier with SPIOs.


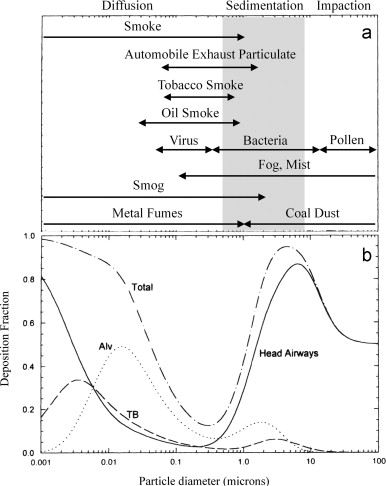


Suppl. Fig. 3 ICRP deposition statistics.
